# Crystal structure of 2-methyl-1*H*-imidazol-3-ium aqua­tri­chlorido­(oxalato-κ^2^ *O*,*O*′)stannate(IV)

**DOI:** 10.1107/S2056989015005988

**Published:** 2015-04-22

**Authors:** Mouhamadou Birame Diop, Libasse Diop, Laurent Plasseraud, Thierry Maris

**Affiliations:** a Laboratoire de Chimie Minérale et Analytique, Département de Chimie, Faculté des Sciences et Techniques, Université Cheikh Anta Diop, Dakar, Senegal; b ICMUB UMR 6302, Université de Bourgogne, Faculté des Sciences, 9 avenue Alain Savary, 21000 Dijon, France; c Département de Chimie, Université de Montréal, 2900 Boulevard Édouard-Montpetit, Montréal, Québec, H3C 3J7, Canada

**Keywords:** crystal structure, organotin(IV) complex, hydrogen bonds

## Abstract

The tin(IV) atom in the complex anion of the title salt, (C_4_H_7_N_2_)[Sn(C_2_O_4_)Cl_3_(H_2_O)], is in a distorted octa­hedral coordination environment defined by three chlorido ligands, an oxygen atom from a water mol­ecule and two oxygen atoms from a chelating oxalate anion. The organic cation is linked through a bifurcated N—H⋯O hydrogen bond to the free oxygen atoms of the oxalate ligand of the complex [Sn(H_2_O)Cl_3_(C_2_O_4_)]^−^ anion. Neighbouring stannate(IV) anions are linked through O—H⋯O hydrogen bonds involving the water mol­ecule and the two non-coordinating oxalate oxygen atoms. In combination with additional N—H⋯Cl hydrogen bonds between cations and anions, a three-dimensional network is spanned.

## Abstract 2

N—H⋯O, N—H⋯Cl and O—H⋯O hydrogen bonds between cations and anions in the complex salt (C_4_H_7_N_2_)^+^[Sn(H_2_O)Cl_3_(C_2_O_4_)]^−^ are responsible for the formation of a three-dimensional network structure.

## Chemical Context   

With many applications found in catalysis (see, for example: Meneghetti & Meneghetti, 2015 ▸) or as a result of their biological activities (Sirajuddin *et al.*, 2014 ▸), organotin(IV) complexes are still a widely studied class of compounds. For more than two decades, the Senegalese group has focused research on attempts to obtain new halo- and organotin(IV) compounds, especially compounds with oxalato ligands (Gueye *et al.*, 2010 ▸, 2012 ▸, 2014 ▸; Sarr *et al.*, 2015 ▸; Sow *et al.*, 2012 ▸, 2013 ▸).
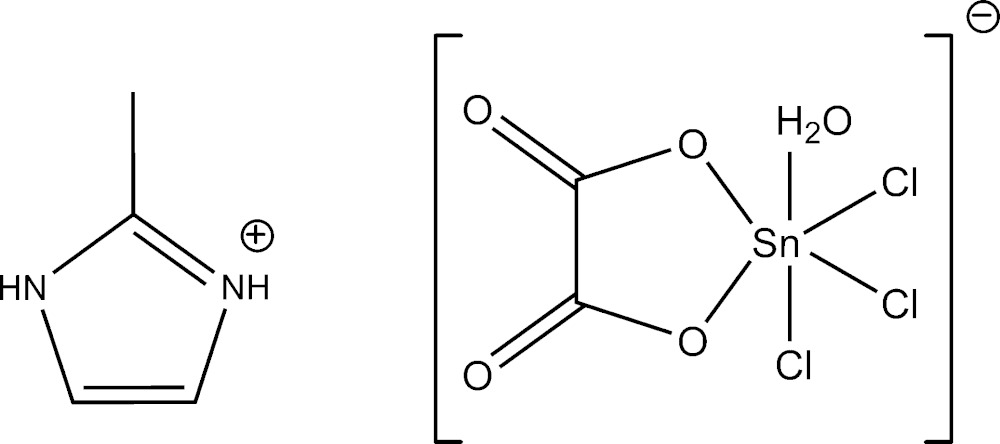


In this communication we report on the inter­action between methyl-2-imidazolium hydrogenoxalate dihydrate and SnCl_2_·2H_2_O in methano­lic solution, which yielded the title compound, (C_4_H_7_N_2_)[Sn(C_2_O_4_)Cl_3_(H_2_O)].

## Structural commentary   

**Figure 1:**
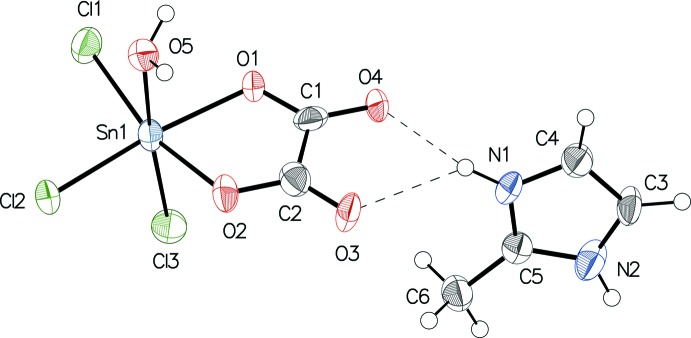
The mol­ecular components of the title compound, with atom labels and 50% displacement ellipsoids at the 50% probability level. Hydrogen atoms are drawn as spheres of arbitrary radius.

The oxalate anion chelates the [SnCl_3_(H_2_O)]^+^ moiety and completes a distorted octa­hedral environment around the tin(IV) atom in the anion (Fig. 1 ▸). The Sn—Cl distances [2.359 (2)–2.378 (3) Å] and the Sn—O distances [2.097 (6) Å and 2.111 (6) Å] are similar to those reported for the same anion in ((H_3_C)_4_N)[Sn(H_2_O)Cl_3_(C_2_O_4_)] (Sow *et al.*, 2013 ▸). The pairwise distribution of C—O bond lengths with two shorter [1.235 (12)/1.243 (12) Å for O3/O4] and two longer bonds [1.277 (11)/1.282 (12) Å for O1/O2] is attributed to additional bonding to the Sn^IV^ atom for the longer bonds. The water mol­ecule is *trans* to one of the Cl atoms and the Sn—O5 bond linking the water mol­ecule to the tin(IV) atom [2.124 (7) Å] is slightly longer than the Sn—O bonds involving the oxalate O atoms. The angles in the [Sn(H_2_O)Cl_3_(C_2_O_4_)]^−^ anion and in the organic cation have typical values.

## Supra­molecular features   

**Figure 2:**
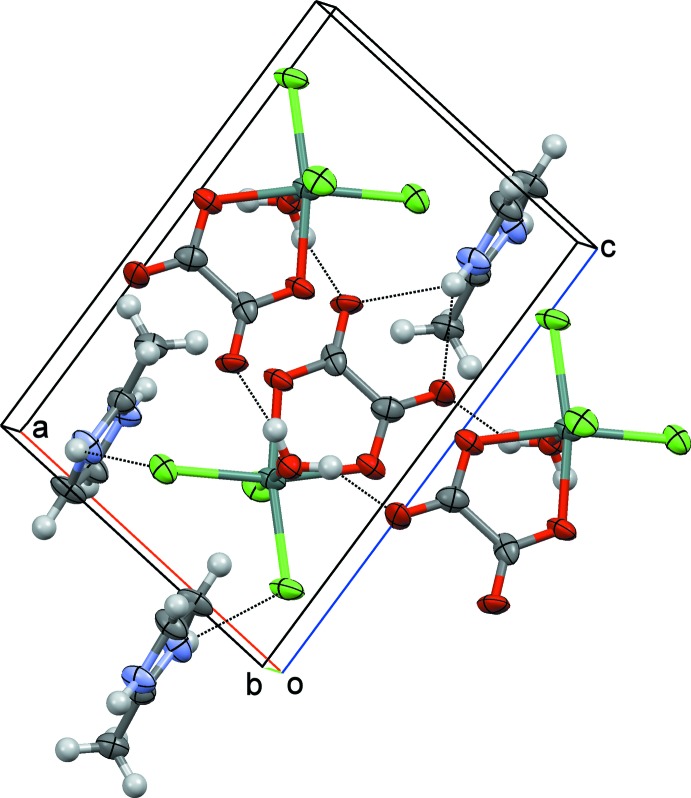
View approximately around the *b* axis showing a central complex anion acting as a hydrogen-bond donor toward two other anions and as a hydrogen-bond acceptor of three methyl-2-imidazolium cations.

**Figure 3:**
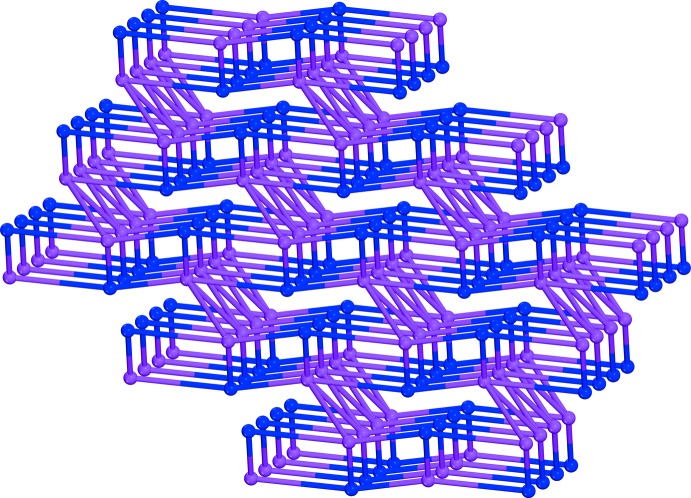
The 3,5T1 topological network in the structure of the title compound. The purple nodes correspond to the Sn^IV^ atoms while the blue nodes are the centres of the organic cations.

Each complex [Sn(H_2_O)Cl_3_(C_2_O_4_)]^−^ anion is linked with two other anions through O—H⋯O hydrogen bonds between the water mol­ecules as donor and non-coordinating oxalate O atoms as acceptor groups (Table 1 ▸). The cations are connected to the anions through a bifurcated N—H⋯O hydrogen bond. Additional N—H⋯Cl hydrogen bonding between cations and anions stabilizes this three-dimensional arrangement (Table 1 ▸, Fig. 2 ▸). Topological analysis according to *TOPOS* (Alexandrov *et al.*, 2011 ▸) reveals a net with 3,5T1 topological type (Fig. 3 ▸).

## Database Survey   

A search of the Cambridge Structural Database (Version 5.36 with one update, Groom & Allen, 2014 ▸) returned about 50 different structures with bidentate oxalate anions linked to a Sn^IV^ atom, from which 23 have their oxalate anions acting as bridging ligands, while 20 have the same configuration as in the title compound with a pairwise distribution of C—O bond lengths. Four structures include both configurations, see, for example: Gueye *et al.* (2010 ▸) or Ng *et al.* (1992 ▸).

## Synthesis and crystallization   

Crystals of methyl-2-imidazolium hydrogenoxalate dihydrate (*L*) were obtained by mixing methyl-2-imidazole with oxalic acid in a 1:1 ratio in water and evaporation of the solvent at 333 K. On allowing (*L*) to react with SnCl_2_·2H_2_O in a 1:2 ratio in methanol, crystals of (C_4_H_7_N_2_)^+^[Sn(H_2_O)Cl_3_(C_2_O_4_)]^−^ were obtained after slow solvent evaporation at room temperature.

## Refinement   

Crystal data, data collection and structure refinement details are summarized in Table 2 ▸. H atoms of the water mol­ecules were obtained from a difference map and were refined with an O—H distance of 0.87 Å and *U*_iso_(H) = 1.5*U*_eq_(O). The other H atoms were positioned geometrically (C—H = 0.95 for aromatic and 0.98 Å for methyl groups; N—H = 0.88 Å) and refined as riding with *U*_iso_(H) = *xU*_eq_(C,N) with *x* = 1.5 for methyl and *x* = 1.2 for all other H atoms.

## Supplementary Material

Crystal structure: contains datablock(s) I, global. DOI: 10.1107/S2056989015005988/wm5136sup1.cif

Structure factors: contains datablock(s) I. DOI: 10.1107/S2056989015005988/wm5136Isup2.hkl

CCDC reference: 1056053

Additional supporting information:  crystallographic information; 3D view; checkCIF report

## Figures and Tables

**Table 1:** Hydrogen-bond geometry (, )

*D*H*A*	*D*H	H*A*	*D* *A*	*D*H*A*
O5H5*A*O4^i^	0.87	1.76	2.618(9)	170
O5H5*B*O3^ii^	0.87	1.83	2.602(9)	146
N1H1O3	0.88	2.32	3.010(11)	136
N1H1O4	0.88	2.31	2.974(10)	132
N2H2Cl2^iii^	0.88	2.70	3.354(8)	132
N2H2Cl1^iv^	0.88	2.84	3.435(10)	126

Symmetry codes: (i) 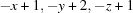; (ii) 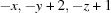; (iii) 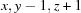; (iv) 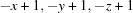.

**Table 2:** Experimental details

Crystal data	
Chemical formula	(C_4_H_7_N_2_)[Sn(C_2_O_4_)Cl_3_(H_2_O)]
*M*_r_	414.19
Crystal system, space group	Triclinic, *P* 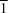
Temperature (K)	120
*a*, *b*, *c* ()	7.4757(9), 8.0857(10), 11.2846(14)
, , ()	80.856(8), 83.946(9), 86.587(8)
*V* (^3^)	669.05(14)
*Z*	2
Radiation type	Ga *K*, = 1.34139
(mm^1^)	13.92
Crystal size (mm)	0.05 0.04 0.04

Data collection	
Diffractometer	Bruker Venture Metaljet
Absorption correction	Multi-scan (*SADABS*; Krause *et al.*, 2015 ▸)
*T*_min_, *T*_max_	0.133, 0.255
No. of measured, independent and observed [*I* > 2(*I*)] reflections	5497, 2520, 1604
*R*_int_	0.112
(sin /)_max_ (^1^)	0.619

Refinement	
*R*[*F* ^2^ > 2(*F* ^2^)], *wR*(*F* ^2^), *S*	0.062, 0.150, 1.07
No. of reflections	2520
No. of parameters	156
H-atom treatment	H-atom parameters constrained
_max_, _min_ (e ^3^)	2.10, 1.23

Computer programs: *APEX2* and *SAINT* (Bruker, 2014 ▸), *SHELXT* (Sheldrick, 2015*a* ▸), *SHELXL2014* (Sheldrick, 2015*b* ▸), *OLEX2* (Dolomanov *et al.*, 2009 ▸), *Mercury* (Macrae *et al.*, 2008 ▸) and *publCIF* (Westrip, 2010 ▸).

## supplementary crystallographic information

### Crystal data

**Table:**

(C_4_H_7_N_2_)[Sn(C_2_O_4_)Cl_3_(H_2_O)]	*Z* = 2
*M**_r_* = 414.19	*F*(000) = 400
Triclinic, *P*1	*D*_x_ = 2.056 Mg m^−^^3^
*a* = 7.4757 (9) Å	Ga *K*α radiation, λ = 1.34139 Å
*b* = 8.0857 (10) Å	Cell parameters from 2537 reflections
*c* = 11.2846 (14) Å	θ = 3.5–53.3°
α = 80.856 (8)°	µ = 13.92 mm^−^^1^
β = 83.946 (9)°	*T* = 120 K
γ = 86.587 (8)°	Block, clear light colourless
*V* = 669.05 (14) Å^3^	0.05 × 0.04 × 0.04 mm

### Data collection

**Table:**

Bruker Venture Metaljet diffractometer	2520 independent reflections
Radiation source: Metal Jet, Gallium Liquid Metal Jet Source	1604 reflections with *I* > 2σ(*I*)
Helios MX Mirror Optics monochromator	*R*_int_ = 0.112
Detector resolution: 10.24 pixels mm^-1^	θ_max_ = 56.1°, θ_min_ = 4.8°
ω and φ scans	*h* = −8→9
Absorption correction: multi-scan (*SADABS*; Krause *et al.*, 2015)	*k* = −9→9
*T*_min_ = 0.133, *T*_max_ = 0.255	*l* = −12→13
5497 measured reflections	

### Refinement

**Table:**

Refinement on *F*^2^	Primary atom site location: structure-invariant direct methods
Least-squares matrix: full	Hydrogen site location: mixed
*R*[*F*^2^ > 2σ(*F*^2^)] = 0.062	H-atom parameters constrained
*wR*(*F*^2^) = 0.150	*w* = 1/[σ^2^(*F*_o_^2^) + (0.0517*P*)^2^ + 1.7851*P*] where *P* = (*F*_o_^2^ + 2*F*_c_^2^)/3
*S* = 1.07	(Δ/σ)_max_ < 0.001
2520 reflections	Δρ_max_ = 2.10 e Å^−^^3^
156 parameters	Δρ_min_ = −1.23 e Å^−^^3^
0 restraints	

### Special details

**Table:**

Experimental. X-ray crystallographic data for I were collected from a single-crystal sample, which was mounted on a loop fiber. Data were collected using a Bruker Venture diffractometer equipped with a Photon 100 CMOS Detector, a Helios MX optics and a Kappa goniometer. The crystal-to-detector distance was 4.0 cm, and the data collection was carried out in 1024 *x* 1024 pixel mode.
Geometry. All e.s.d.'s (except the e.s.d. in the dihedral angle between two l.s. planes) are estimated using the full covariance matrix. The cell e.s.d.'s are taken into account individually in the estimation of e.s.d.'s in distances, angles and torsion angles; correlations between e.s.d.'s in cell parameters are only used when they are defined by crystal symmetry. An approximate (isotropic) treatment of cell e.s.d.'s is used for estimating e.s.d.'s involving l.s. planes.

### Fractional atomic coordinates and isotropic or equivalent isotropic displacement parameters (Å^2^)

**Table:**

	*x*	*y*	*z*	*U*_iso_*/*U*_eq_	
Sn1	0.29887 (9)	0.93269 (8)	0.27360 (5)	0.0317 (2)	
Cl2	0.0743 (4)	1.0116 (3)	0.1403 (2)	0.0435 (6)	
Cl3	0.3305 (4)	0.6485 (3)	0.2412 (2)	0.0440 (6)	
Cl1	0.5565 (3)	1.0303 (3)	0.1490 (2)	0.0408 (6)	
O1	0.4545 (9)	0.8696 (8)	0.4197 (5)	0.0350 (16)	
O5	0.2658 (9)	1.1790 (8)	0.3182 (6)	0.0353 (16)	
H5A	0.3590	1.2042	0.3507	0.053*	
H5B	0.1714	1.1866	0.3693	0.053*	
O3	0.0676 (9)	0.7477 (9)	0.6101 (6)	0.0382 (16)	
O2	0.0984 (9)	0.8685 (8)	0.4173 (5)	0.0346 (15)	
O4	0.4338 (9)	0.7392 (9)	0.6108 (6)	0.0384 (17)	
N1	0.2275 (12)	0.5715 (10)	0.8327 (7)	0.042 (2)	
H1	0.2288	0.6585	0.7749	0.050*	
C1	0.3663 (13)	0.8049 (12)	0.5176 (9)	0.035 (2)	
C5	0.2088 (13)	0.4176 (12)	0.8164 (9)	0.034 (2)	
C2	0.1599 (14)	0.8079 (13)	0.5178 (9)	0.038 (2)	
N2	0.2132 (12)	0.3254 (11)	0.9248 (7)	0.046 (2)	
H2	0.2019	0.2162	0.9399	0.055*	
C3	0.2380 (15)	0.4238 (12)	1.0101 (9)	0.041 (3)	
H3	0.2481	0.3874	1.0935	0.050*	
C4	0.2450 (16)	0.5808 (14)	0.9517 (9)	0.044 (3)	
H4	0.2592	0.6791	0.9854	0.053*	
C6	0.1870 (14)	0.3569 (13)	0.7014 (9)	0.040 (2)	
H6A	0.0680	0.3939	0.6757	0.060*	
H6B	0.1982	0.2342	0.7134	0.060*	
H6C	0.2804	0.4026	0.6393	0.060*	

### Atomic displacement parameters (Å^2^)

**Table:**

	*U*^11^	*U*^22^	*U*^33^	*U*^12^	*U*^13^	*U*^23^
Sn1	0.0409 (4)	0.0320 (4)	0.0223 (4)	−0.0033 (3)	−0.0078 (2)	−0.0003 (3)
Cl2	0.0552 (15)	0.0464 (15)	0.0291 (13)	−0.0117 (12)	−0.0202 (11)	0.0076 (12)
Cl3	0.0584 (15)	0.0350 (14)	0.0407 (14)	−0.0062 (12)	−0.0077 (12)	−0.0090 (12)
Cl1	0.0482 (13)	0.0417 (14)	0.0302 (12)	−0.0063 (11)	0.0016 (11)	−0.0003 (11)
O1	0.049 (4)	0.037 (4)	0.020 (3)	−0.012 (3)	−0.010 (3)	0.004 (3)
O5	0.038 (4)	0.039 (4)	0.029 (4)	−0.006 (3)	−0.003 (3)	−0.005 (3)
O3	0.037 (4)	0.042 (4)	0.029 (4)	0.002 (3)	−0.001 (3)	0.014 (3)
O2	0.049 (4)	0.034 (4)	0.021 (3)	0.005 (3)	−0.005 (3)	−0.004 (3)
O4	0.041 (4)	0.042 (4)	0.031 (4)	−0.007 (3)	−0.020 (3)	0.012 (3)
N1	0.061 (6)	0.029 (5)	0.033 (5)	−0.013 (4)	−0.011 (4)	0.010 (4)
C1	0.046 (6)	0.030 (5)	0.033 (6)	−0.018 (5)	0.005 (5)	−0.010 (5)
C5	0.038 (5)	0.033 (5)	0.032 (5)	−0.003 (4)	−0.005 (4)	−0.003 (5)
C2	0.048 (6)	0.031 (6)	0.037 (6)	0.002 (5)	−0.012 (5)	−0.005 (5)
N2	0.066 (6)	0.032 (5)	0.035 (5)	0.000 (4)	0.002 (4)	0.003 (4)
C3	0.072 (7)	0.025 (5)	0.026 (5)	0.004 (5)	−0.012 (5)	0.003 (5)
C4	0.068 (7)	0.035 (6)	0.031 (6)	−0.011 (5)	−0.005 (5)	−0.007 (5)
C6	0.049 (6)	0.038 (6)	0.035 (6)	0.001 (5)	−0.012 (5)	−0.004 (5)

### Geometric parameters (Å, º)

**Table:**

Sn1—Cl2	2.364 (3)	N1—C5	1.304 (13)
Sn1—Cl3	2.378 (3)	N1—C4	1.377 (12)
Sn1—Cl1	2.359 (2)	C1—C2	1.542 (14)
Sn1—O1	2.097 (6)	C5—N2	1.330 (12)
Sn1—O5	2.124 (7)	C5—C6	1.486 (13)
Sn1—O2	2.111 (6)	N2—H2	0.8800
O1—C1	1.277 (11)	N2—C3	1.375 (12)
O5—H5A	0.8700	C3—H3	0.9500
O5—H5B	0.8691	C3—C4	1.336 (14)
O3—C2	1.235 (12)	C4—H4	0.9500
O2—C2	1.282 (12)	C6—H6A	0.9800
O4—C1	1.243 (12)	C6—H6B	0.9800
N1—H1	0.8800	C6—H6C	0.9800
			
Cl2—Sn1—Cl3	95.47 (10)	O4—C1—O1	125.3 (9)
Cl1—Sn1—Cl2	100.40 (9)	O4—C1—C2	118.3 (8)
Cl1—Sn1—Cl3	97.56 (9)	N1—C5—N2	105.5 (8)
O1—Sn1—Cl2	168.07 (19)	N1—C5—C6	127.6 (9)
O1—Sn1—Cl3	88.82 (19)	N2—C5—C6	126.9 (9)
O1—Sn1—Cl1	90.03 (18)	O3—C2—O2	125.0 (10)
O1—Sn1—O5	87.8 (3)	O3—C2—C1	119.3 (9)
O1—Sn1—O2	78.6 (3)	O2—C2—C1	115.6 (9)
O5—Sn1—Cl2	87.18 (19)	C5—N2—H2	124.6
O5—Sn1—Cl3	175.21 (18)	C5—N2—C3	110.9 (8)
O5—Sn1—Cl1	85.86 (18)	C3—N2—H2	124.6
O2—Sn1—Cl2	90.23 (19)	N2—C3—H3	127.0
O2—Sn1—Cl3	90.25 (19)	C4—C3—N2	106.0 (9)
O2—Sn1—Cl1	166.09 (18)	C4—C3—H3	127.0
O2—Sn1—O5	85.7 (2)	N1—C4—H4	126.9
C1—O1—Sn1	114.2 (6)	C3—C4—N1	106.1 (10)
Sn1—O5—H5A	110.8	C3—C4—H4	126.9
Sn1—O5—H5B	110.3	C5—C6—H6A	109.5
H5A—O5—H5B	108.2	C5—C6—H6B	109.5
C2—O2—Sn1	114.3 (6)	C5—C6—H6C	109.5
C5—N1—H1	124.2	H6A—C6—H6B	109.5
C5—N1—C4	111.5 (9)	H6A—C6—H6C	109.5
C4—N1—H1	124.2	H6B—C6—H6C	109.5
O1—C1—C2	116.5 (9)		
			
Sn1—O1—C1—O4	170.5 (8)	N1—C5—N2—C3	−0.9 (12)
Sn1—O1—C1—C2	−8.7 (10)	C5—N1—C4—C3	0.6 (13)
Sn1—O2—C2—O3	−172.8 (8)	C5—N2—C3—C4	1.3 (13)
Sn1—O2—C2—C1	4.3 (10)	N2—C3—C4—N1	−1.1 (13)
O1—C1—C2—O3	−179.7 (8)	C4—N1—C5—N2	0.2 (12)
O1—C1—C2—O2	3.0 (13)	C4—N1—C5—C6	−179.8 (10)
O4—C1—C2—O3	1.1 (14)	C6—C5—N2—C3	179.1 (10)
O4—C1—C2—O2	−176.2 (8)		

### Hydrogen-bond geometry (Å, º)

**Table:**

*D*—H···*A*	*D*—H	H···*A*	*D*···*A*	*D*—H···*A*
O5—H5*A*···O4^i^	0.87	1.76	2.618 (9)	170
O5—H5*B*···O3^ii^	0.87	1.83	2.602 (9)	146
N1—H1···O3	0.88	2.32	3.010 (11)	136
N1—H1···O4	0.88	2.31	2.974 (10)	132
N2—H2···Cl2^iii^	0.88	2.70	3.354 (8)	132
N2—H2···Cl1^iv^	0.88	2.84	3.435 (10)	126

Symmetry codes: (i) −*x*+1, −*y*+2, −*z*+1; (ii) −*x*, −*y*+2, −*z*+1; (iii) *x*, *y*−1, *z*+1; (iv) −*x*+1, −*y*+1, −*z*+1.
